# Mapping female bodily features of attractiveness

**DOI:** 10.1038/srep18551

**Published:** 2016-01-21

**Authors:** Jeanne Bovet, Junpeng Lao, Océane Bartholomée, Roberto Caldara, Michel Raymond

**Affiliations:** 1 Institute for Advanced Study in Toulouse, Manufacture des Tabacs, 21 allée de Brienne, 31015 Toulouse Cedex 6, France; 2 Department of Psychology, University of Fribourg, Switzerland; 3 Institute of Evolutionary Sciences, University of Montpellier, CNRS, IRD, EPHE, France

## Abstract

“Beauty is bought by judgment of the eye” (Shakespeare, Love’s Labour’s Lost), but the bodily features governing this critical biological choice are still debated. Eye movement studies have demonstrated that males sample coarse body regions expanding from the face, the breasts and the midriff, while making female attractiveness judgements with natural vision. However, the visual system ubiquitously extracts diagnostic extra-foveal information in natural conditions, thus the visual information actually used by men is still unknown. We thus used a parametric gaze-contingent design while males rated attractiveness of female front- and back-view bodies. Males used extra-foveal information when available. Critically, when bodily features were only visible through restricted apertures, fixations strongly shifted to the hips, to potentially extract hip-width and curvature, then the breast and face. Our hierarchical mapping suggests that the visual system primary uses hip information to compute the waist-to-hip ratio and the body mass index, the crucial factors in determining sexual attractiveness and mate selection.

Behavioral studies have suggested that body fat (estimated by the body-mass index: BMI) and its distribution (estimated by the waist-to-hip ratio: WHR) play a critical role in the evaluation of female physical attractiveness by men^1^,^2^,^3^,^4^,^5^,^6^,^7^,^8^,^9^. However, none of these studies could directly map out the bodily visual information used by the observers to *actively* achieve this crucial biological categorization for mating, as well as their respective importance in determining this choice. More recently, eye-tracking studies have attempted to address this question by analyzing observers’ fixation patterns of morphological traits while performing attractiveness judgments^10^,^11^,^12^,^13^,^14^. These studies have shown that when men were evaluating the attractiveness of female bodies, much of their attention was directed to the face, breasts and midriff. Typically, these eye movement studies relied on the use of a Region or Area of Interest (ROI or AOI) approach for statistical analyses, which reduce the high-dimensional visual input space (thousands or millions of pixels of the presented image) into a low-dimensional space. Although ROIs are implicitly assumed to optimally represent the visual categories present in the visual input space (e.g., the face, breasts, midriff and leg regions), they suffer from an arbitrary and *subjective* segmentation of the visual inputs into ROIs, which are constrained by the a priori biases of the experimental framework and experimenters^15^. In the present context, ROIs used in attractiveness studies are thus often over-represented, and their boundaries are difficult to determine (e.g., the midriff area extends from the below the breasts to the widest part of the hip^13^,^14^). This shortcoming leads to a considerable loss of spatial resolution, with arbitrary categorization of eye movements falling on the ROIs’ borders and the loss of subtle patterns of eye movements between ROIs^10^,^11^,^13^,^14^. Another critical drawback of previous eye movement studies is their reliance on the use of natural vision paradigms for determining fine-grained visual information use, because the visual system can extract diagnostic information without focal fixations. For instance, Kuhn and Tatler^16^ have shown that people can detect the misdirection in a magic performance without fixating on the precise location where the trick is taking place. Similar findings demonstrate that information extracted from a foveal fixation during natural viewing does not straightforwardly translate to information use. Thus men could use visual information located in body regions which are not directly fixated during attractiveness assessments.

To address this question, we recorded the eye movements of male observers during attractiveness judgments by combining a data-driven analysis (i.e., *i*Map4^15^,^17^) and a gaze-contingent design^18^. *i*Map4 implements a Linear Mixed-Model (LMM – see methods) with a robust statistical approach to correct for the multiple comparison problem driven by repetitive testing in pixel space. *i*Map4 was thus used to *objectively* define the regions of the body fixated during men’s assessment of female attractiveness; no a priori image segmentation is required with this technique. To finely estimate information use, we used a conventional natural viewing paradigm and also gaze-contingent paradigms in which the stimulus display is continuously updated as a function of the observers’ current gaze position. We parametrically restricted the bodily information available to the observers by using ‘*Spotlights*’ with 2° (foveal vision only) or 4° apertures dynamically centered on observers’ fixations to isolate information use (see Fig. 1). Crucially, the 2° Spotlight apertures covered a single bodily feature (i.e., face, breast or hip), while the 4° condition was closer to natural viewing conditions: information from the outline of the body could be available to the observers when they fixed at the center of the torso. Participants rated the attractiveness of front- and back-view computer-morphed women’s bodies (see Fig. 2) under these three viewing conditions.

Concordant with previous studies, attractiveness ratings were influenced by face^19^,^20^, WHR^1^,^2^,^3^,^4^,^5^,^6^,^7^,^8^,^9^ and hip size^21^,^22^. As expected, observers directed their gaze towards the women’s stomach and to the central part of the chest (sternum) during natural vision, corroborating previous findings^12^. However, these physical features (sternum and stomach) are not known, in the literature, to influence female attractiveness. When the front-view body images were visible only through a restricted aperture (*Spotlights*), the observers’ fixations were still directed to the women’s face/head, but also progressively shifted to the breasts and the hips. Importantly, the hip area was the only bodily feature invariantly used to perform judgments of attractiveness with front- and back-view bodies, showing the largest fixation increase among the critical features involved in physical attractiveness. This observation posits the hips as being the critical diagnostic region for evaluating female attractiveness.

## Results

### Behavioral analyses

The results from the general linear mixed model showed that the viewing condition had a significant effect on attractiveness ratings (*β* = 0.90 [0.80; 1] for the *4*° *spotlight* and *β* = 1.03 [0.93; 1.13] for *2*° *spotlight*, *p* < 0.0001, see supplementary table S1; square brackets report the 95% confidence interval): the more restricted the peripheral vision, the higher the ratings. The results from the linear mixed models according to the viewing conditions showed a significant negative relationship between WHR and attractiveness ratings in both *natural viewing* and *4*° *spotlight* conditions (*β*_*natural viewing*_ = −5.78 [−7.43; −4.13] and *β*_*4*° *spotlight*_ = −3.77 [−5.28; −2.27], *p* < 0.0001, respectively, see supplementary table S2). However, the relationship between the two was not significant in the *2*° *spotlight* condition (*β*_*2*° *spotlight*_ = −0.83 [−2.33; 0.65], *p* = 0.30). Observers rated female bodies with a low WHR more favorably when they had access to sufficient peripheral vision. The hip size had a significant negative effect on the attractiveness ratings for all the conditions (*β*_*natural viewing*_ = −0.13 [−0.14; −0.11], *β*_*4*° *spotlight*_ = −0.05 [−0.06; −0.03] and *β*_*2*° *spotlight*_ = −0.02 [−0.03; −0.007], *p* < 0.006). Female bodies with overly large hip sizes received a lower attractiveness rating regardless of *viewing conditions*. The face also significantly influenced the subjective ratings for all the conditions (*p* < 0.0001). The effect of the female body’s orientation (front or back) was significant for the *natural viewing* and *2*° *spotlight* conditions (*β*_*natural viewing*_ = 0.16 [0.01; 0.31] and *β*_*2*° *spotlight*_ = −0.14 [−0.28; −0.01], *p* = 0.045 for both), but not for the *4*° *spotlight* condition (*β*_*4*° *spotlight*_ = 0.07 [−0.06; 0.21], *p* = 0.27). Men’s preference for the back view (compared to the front view) under *natural viewing* disappeared when the peripheral vision was restricted through the *4*° *spotlight*, and changed for a preference for the front view for the *2*° *spotlight* condition. None of the observers’ characteristics (age, monthly income, or education level) showed modulation on their ratings (*p* > 0.05).

### Eye movements

Overall participants fixated longer during spotlight conditions: front view (*2*° *spotlight* M = 16.22 s, SD = 9.45; *4*° *spotlight* M = 8.77 s, SD = 4.20; *natural viewing* M = 5.28 s, SD = 3.68), back view (*2*° *spotlight* M = 15.36 s, SD = 8.70; *4*° *spotlight* M = 8.21 s, SD = 3.29; *natural viewing* M = 4.27 s, SD = 2.79). To investigate the fine-grained differences in the fixation pattern across conditions, we fitted two LMMs with a data-driven approach using *i*Map4. After the initial model fitting, pixel-wise ANOVA on the Linear Mixed-Model with a full design (LMM – equation 1 in the methods section) revealed significant main effects for both viewing condition and body orientation (see supplementary figure S1) after using a bootstrap clustering procedure to correct for multiple comparisons. The interaction did not reach significance. Importantly, the main effect of the viewing condition is shown around the hips (Peak: F (2,195) = 45.72, minimal: F (2,195) = 18.99, p < 0.05 cluster corrected), while a significant effect of the body orientation is clustered around the face region (Peak: F (1,195) = 119.01, minimal: F (1,195) = 33.46, p < 0.05 cluster corrected). The main effect of subjective attractiveness ratings was not significant, suggesting that there is no obvious relationship between the time spent on the body areas fixated and the judgments of attractiveness.

To further clarify these findings, we estimated the coefficients for each categorical predictor on the LMM with a categorical model (see equation 2 in methods section). As shown in Fig. 3, observers fixated on the face region when the body was present in the full front orientation, regardless of the visual field size (local maximum on the face area in front view condition: *β*_*natural viewing*_ = 273.8 [107.7, 445.9], *β*_*4*° *spotlight*_ = 461.5 [284.5, 638.5], *β*_*2*° *spotlight*_ = 639.7 [465.1, 814.4]). As a comparison, the hip areas were fixated longer in the 2° spotlight condition regardless of the body orientation (local maximum of 2° spotlight condition: *β*_*front view*_ = 228.2 [169.7, 286.8], *β*_*back view*_ = 273.3 [214.7, 338.8]). Finally, to obtain a fine-grained picture of information use, we performed a pixel-wise linear contrast between the 2° spotlight condition and the natural viewing conditions (see Fig. 4 left panel, contrast test F-value at peak: F (1,195) = 79.10, minimal: F (1,195) = 33.42, p < 0.05 cluster corrected). This analysis revealed that the upper part of the face, the breast and the hip areas are the key bodily features sampled for performing attractiveness judgments. To further quantify the relative importance of information among these areas, we computed post-hoc analysis within each significant area (i.e., face, breast and hip area) from the linear contrast. We computed the normalized contrast of any two given front-view conditions by dividing the contrast of the peak values by their sum within each body area. As shown in the right panel of Fig. 4, the weight contrast from the hip area changed the most rapidly as compared to other body regions (F (2,285) = 19.249, p < 0.05).

## Discussion

The aim of the experiment was to identify the information actively used by the participants when assessing female body attractiveness, and we show that men use their peripheral vision for this assessment. The gaze-contingent aperture size significantly affected the way men look at female bodies: when observers could access visual information only through a narrow aperture, they gazed more at the exterior edges of the torso – the hips – (front and back view) and the breast (front view), while during natural viewing they favored the center of these regions as they could process the entire silhouette from this location.

The viewing condition also affected the attractiveness ratings, as observers’ ratings increased when the peripheral vision was increasingly restricted. Moreover, the back view was preferred over the front view during natural viewing, but this effect reversed when vision was restricted to foveal vision (spotlight with 2° of visual angle) and both hips could not be perceived with a single fixation. This observation favors the view that the hips region might play a critical role in attractive ratings. However, this result could also be due to the nature of the stimuli we used. Given that in the front view condition the observers were processing multiple body features simultaneously, the artificial nature of the stimuli might have decreased the attractiveness ratings compared to the back view condition (displaying mainly a single body region of interest). Regardless of both explanations, further experiments using real women or body parts shown separately are necessary to further clarify this issue.

Altogether, these results indicate that the information necessary to estimate female attractiveness can be gathered without directly staring at the contours of the hips when peripheral vision is available, but clearly the center of the torso itself is *not* containing diagnostic information. Interestingly, our data also showed the largest increase in fixations towards the hips during the 2° foveal vision condition (for both front-and back-views), followed by the face and the breast with front-views, giving this region a pivotal role in the evaluation of female attractiveness. The information collected in this area could relate to the extraction of the curvature of the hip shape and an estimation of hip width, as highlighted by the fixation patterns with constrained vision. In fact, WHR had an effect on the attractiveness ratings only when the peripheral vision was sufficient (in the *natural viewing* and *4*° *spotlight* conditions), which is concordant with the fact that the whole silhouette is necessary to estimate the WHR^12^.

Why use peripheral vision when relevant information could be extracted directly? Although information is crucial to adaptive decision-making, only a fraction is actually relevant to the task; even so, individuals tend to encounter information at rates considerably higher than their capacity for full use^10^. Attentional resources are necessary for discriminating useful from irrelevant information, but they are limited and so should be allocated selectively. The use of peripheral vision could result in an optimal decision-making system: individuals able to extract only relevant information from the body shape, without amassing superfluous details, will make decisions rapidly and expend less energy in the process. Nevertheless, our study is restricted to a Caucasian population. It is possible that, as for the recognition of faces^18^,^23^, the use of peripheral vision in the assessment of female attractiveness varies between populations. Further investigation is needed to determine the universality–or variability–of men’s eye movement patterns in female attractiveness judgments. Moreover, future studies are necessary to clarify whether male observers would deploy similar eye movement strategies during passive viewing of bodies (i.e. when they are not asked to rate attractiveness).

In line with previous findings, a large number of fixations were directed towards the face in the front-view condition^13^, and to a lesser extent to the head during the back-view condition^14^. Viewing facial features in the front-view had a significant influence on attractiveness ratings^19^,^20^. However, the greatest numbers of fixations were directed towards the hip regions with restricted vision, and their width was the most important determinant of attractiveness, independently from the WHR. This shift toward the external part of the torso was weaker for the waist, confirming that information provided by the hip is more important than the information included at the waist level. Additionally, hip size had a significant effect on attractiveness ratings in all viewing conditions. The importance of hip size for attractiveness, independently of WHR, has been previously reported^21^,^22^. What is the information conveyed by hip size? Large hips could give the impression of a heavier body^4^. Indeed, body width, and especially the lower torso width, is a reliable guide to the BMI^24^. As the hip contours are required to estimate their width, the relevant information of this region (normally provided by the peripheral vision) could be the BMI or a related measure. The BMI is also correlated with health and fertility^25^,^26^,^27^,^28^,^29^. The BMI may be a factor equivalent to -or even more important than- the WHR in determining the physical attractiveness of a woman^21^,^22^,^24^,^30^. However, the design of our study (artificial bodies with varying WHR within a reduced BMI range) does not allow the investigation of this specific point. The extraction of women’s WHR information could have an evolutionary basis because WHR is a reliable indicator of a woman’s age, health and fertility: Compared to women with high WHR, women with a low WHR have fewer irregular menstrual cycles^31^, optimal sex hormone profiles^32^, ovulate more frequently^33^, and have lower endocervical pH, which favors sperm penetration^34^. Low WHR is also an independent predictor of pregnancy in women attending an artificial insemination clinic^35^ and in women attempting *in vitro* embryo fertilization transfer^36^.

In summary, our data show that men use their peripheral vision in judgments of female attractiveness under natural viewing conditions. This could allow for the estimation of the WHR, even without directly staring at the curve of the torso. Additionally, analyses of both attractiveness ratings and eye movements isolated a hierarchy of cue use, positing the hip width as an important feature of female attractiveness, independent from the WHR. The hip width could also be a key feature in estimating the BMI, another trait linked with a woman’s health and fertility. Overall, this bodily feature seems to play a critical role in the evaluation of female attractiveness by men and offers novel insights into the critical visual information beneath the contemporary obsession with being thin.

## Methods

### Stimuli

**Figure 1:**
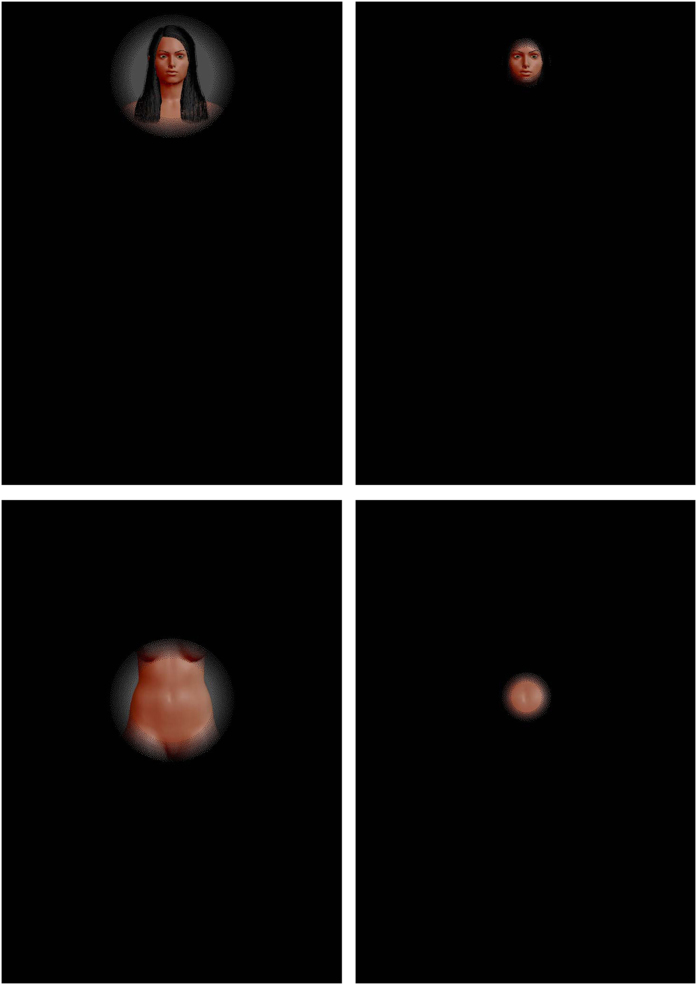
Front views of one stimulus on the two *Spotlight* conditions, with gaze-contingent apertures of 4° (first column) and 2° (second column) centered on the face (first row) and on the navel (second row). The aperture of 2° covered the entire face, but the edges of the torso were not visible when fixating the navel. The aperture 4° allowed one to simultaneously see the edges of both sides of the torso when fixating the navel. Images generated with the software MakeHuman, under a PDD licence (public domain).

We created virtual female bodies with the open source tool MakeHuman, which allows for the flexible morphing of a 3D human body that varies in several dimensions (see Fig. 1). Three waist and 2 hip circumferences were used, resulting in 6 different body shapes representing a relatively large range of WHR (0.67–0.87) within a reduced range of BMI. To avoid an eventual habituation effect, 3 distinct faces (with different haircuts and facial features) were combined with each body shape, resulting in a total of 18 unique virtual female bodies. All other features were kept constant. The WHR of each 3D stimulus was calculated with the software measures, and the BMI was estimated as Volume * Density/(height)^2^, with density = 1.043 g/cm^3 ^37 and the volume (in cm^3^) of each stimulus was measured using the plugin Viewer 3D of ImageJ. Screenshots of female bodies were exported with three different views: front and back, corresponding to a total of 36 images (6 WHR * 3 faces * 2 orientations).

### Participants

Thirty-five male observers participated in the current study. For each observer, the following information was collected: age, monthly income, and education level. As it is known that men’s preferences can change with age^38^, we focused on a narrow age range to control experimentally for this effect. The participants’ ages ranged from 20 to 39 (average 25 years old). The volunteer raters were unaware of the purpose of the study when assessing the artwork. Public advertisements were dispatched in local stores and social networks to recruit volunteers. These advertisements contained the principle of the test (an eye-tracking study on female attractiveness) and our contact information.

### Ethics Statement

All experiments were carried out in accordance with the current laws of France. The protocol used to collect and analyze data was approved (#1226659) by the French National Committee of Information and Liberty (CNIL). For each participant, the general purpose of the study was explained, and written informed consent was obtained from each participant prior to the experiment. Each participant received a compensation of 20€. The data were analyzed anonymously.

### Procedure

**Figure 2:**
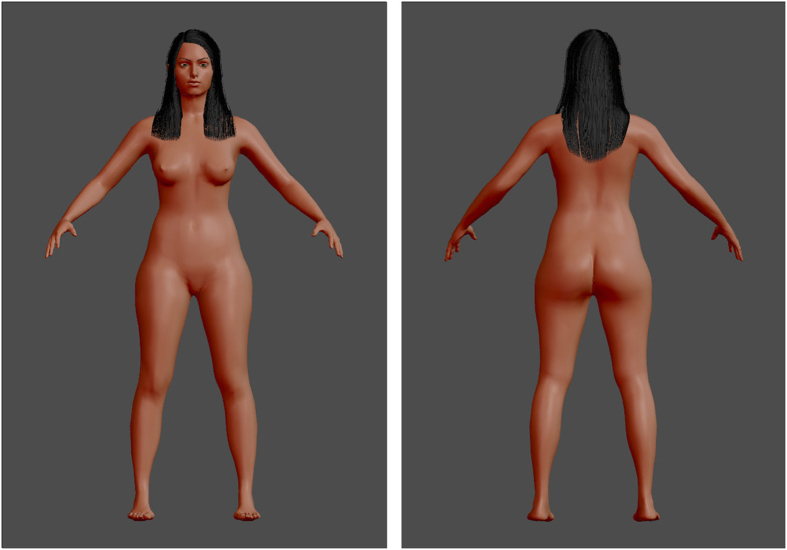
Front- and back-views of a stimulus under the *natural viewing* condition. Images generated with the software MakeHuman, under a PDD licence (public domain).

The experiment was carried out in an isolated room under uniform lighting conditions. The observers were seated in a chair, facing the screen at a distance of 83 cm. The viewing distance was maintained with a forehead and chin rest. The experiment was programmed in SR Research Experiment Builder (version 1.10.1025) and ran on a 3-GHz core i7 computer. The stimuli (924 pixel height) were presented on a 19-inch screen at a 1280 × 1024 resolution with a refresh rate of 60 Hz. The experiment was divided into 3 blocks corresponding to 3 different viewing conditions: a *natural viewing* condition and 2 *spotlight* conditions. For the *natural viewing* condition, the whole picture was displayed (see Fig. 1). For the *spotlight* conditions, the picture was visible through a gaze-contingent circular aperture with a diameter of either 2° or 4° (see Fig. 2). Each body was presented once from a front-view and once from a back-view within each block (see Fig. 1). The aperture edges were blurred, and the visual field outside the aperture was black. The 3 blocks were conveyed in random order for each observer. Within each block, the 36 body images were presented in a random sequence either on the left or the right of the screen to avoid any anticipatory eye movement. The image remained on the screen until the observers pressed the space bar. The participants rated the body attractiveness using a keyboard with an 11-point scale (0 being the least and 10 the most attractive) at the end of each trial. A white dot was presented in the middle of the screen in between each presentation for drift correction. At the beginning of the test, participants were given general instructions concerning the eye-tracker use (position on the forehead and chin rest, instructions for calibration), information about the course of the experiment (“there will be 3 blocks of 36 images, with a break after each block”), and the task was explained (“you will have to rate the attractiveness of each woman, on a 11-point scale. When you are ready, press the space bar to do the rating and pass to the next picture”).

### Eye Tracking

Using the EyeLink® 1000 Desktop Mount system (SR Research Ltd., Ontario, Canada), eye position and eye movements were determined by measuring the corneal reflection and dark pupil with a video-based infrared camera and an infrared reflective mirror. The eye tracker had a spatial resolution of 0.01° of the visual angle, and the signal was sampled and stored at a rate of 1000 Hz. Although the viewing was binocular, the recording was monocular (a standard procedure in eye-tracking studies). Calibration and validation of the measurements were performed before each experimental session.

### Behavioral analyses

We applied a linear mixed model to analyze observers’ subjective ratings. The viewing conditions and the WHR of female bodies were considered to be the main explanatory variables. There were 3 different faces and 2 different hip sizes for each waist size. We thus integrated the face types and hip sizes as additional explanatory variables in the model. The body orientation (front or back) was entered as a confounding effect. Variables concerning the observers’ characteristics (age, monthly income, and education level) were also included in the model as potential confounding effects. Observers were considered as random sample from a larger population of interest and were thus considered as a random-effect variable. Female images were also considered as a random-effect variable to capture potential interactions between the other variables (WHR, hip size, face and body orientation). Overall, our model could be expressed as the following:
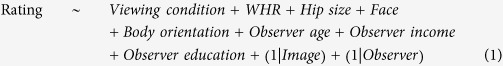


Then, we run this model (without the variable *Viewing condition*) for each viewing condition separately. The linear mixed models were fitted with LMER (package lme4 1.1–9) using R 3.1.1 (R Core Development Team).

### Eye movement analyses

Eye movement data were analyzed using the new version of *iMap*^15^,^17^, which implements a robust data-driven approach based on a Linear Mixed Model (LMM) and a bootstrap clustering method for hypothesis testing. Fixations and saccades were extracted from the raw data by using the default settings in the Eyelink software. Fixation durations were then projected back into the two-dimensional space according to their *x* and *y* coordinates at the single trial level. We smoothed the fixation duration map by convoluting it with a two-dimensional Gaussian Kernel function.
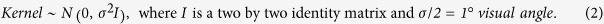


We then estimated the fixation bias of each condition independently for all the observers by taking the expected values across trials within the same condition. The resulting 3D matrix (Conditions * xSize * ySize) was then modeled in a LMM as the response variable. Subjective ratings were also computed by taking the average for each condition. To quantify the spatial bias in the fixation pattern for each experimental manipulation, the intensity of each pixel in the smoothed duration map was modeled with the following LMM specification:
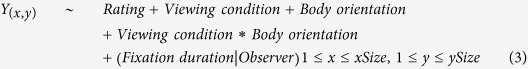


Thus, the fixation duration was considered as a function of *subjective rating*, *viewing condition* (3 levels), *body orientation* (2 levels), and the *interaction between the categorical predictors.* In addition, the mean fixation duration for each condition and the intercept were treated as random effects to control for the variation across individuals. After this analysis, an alternative model without the continuous predictor of subjective rating was also fitted to the fixation maps to better estimate the categorical predictors as follows:
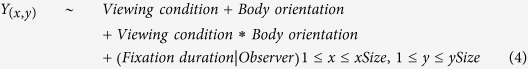


The LMM was fit by maximal likelihood (ML) using the *fitlme* function from the Statistics Toolbox™, Matlab 2013b. Furthermore, the sum of all categorical coefficients in the design matrix were set to zero to allow type III hypothesis testing in analyses of variance (ANOVA). Parametric statistics on the fixed effect and contrasts are performed by the *coefTest* for LinearMixedModel class in the Matlab software. To control for false positives from pixel-wise hypothesis testing, a multiple comparisons correction was applied by using a bootstrap clustering method^39^,^40^. Original parametric statistical values were thresholded at a given *p* value (0.05/the number of pixels). We then calculated the cluster mass by summing the statistic values within each cluster and later compared them with a bootstrap distribution under the null hypothesis. To construct the bootstrap null distribution, the condition mean is removed from each categorical condition and randomized without replacement to disrupt any possible covariation between fixation intensity and attractiveness ratings. This procedure makes sure that H0: *μ* = 0 is true for all potential coefficients while preserving the global variance for the experiment conditions. A subject-wise bootstrap with replacement was then repeated 1000 times to create a set of bootstrap null response matrices. We then performed the same modeling and subsequently the same contrast tests for the selected coefficients across all pixels. The resulting statistical map for each bootstrap was thresholded with the same parameters of the original map. The maximum cluster mass in each bootstrap was put into a vector and then sorted to form a bootstrap distribution for each contrast. Given that bootstrapped response matrices are derived under the null hypothesis, the cluster-wise *p*-value was calculated as:
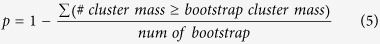
which results in the cluster-corrected p values.

**Figure 3:**
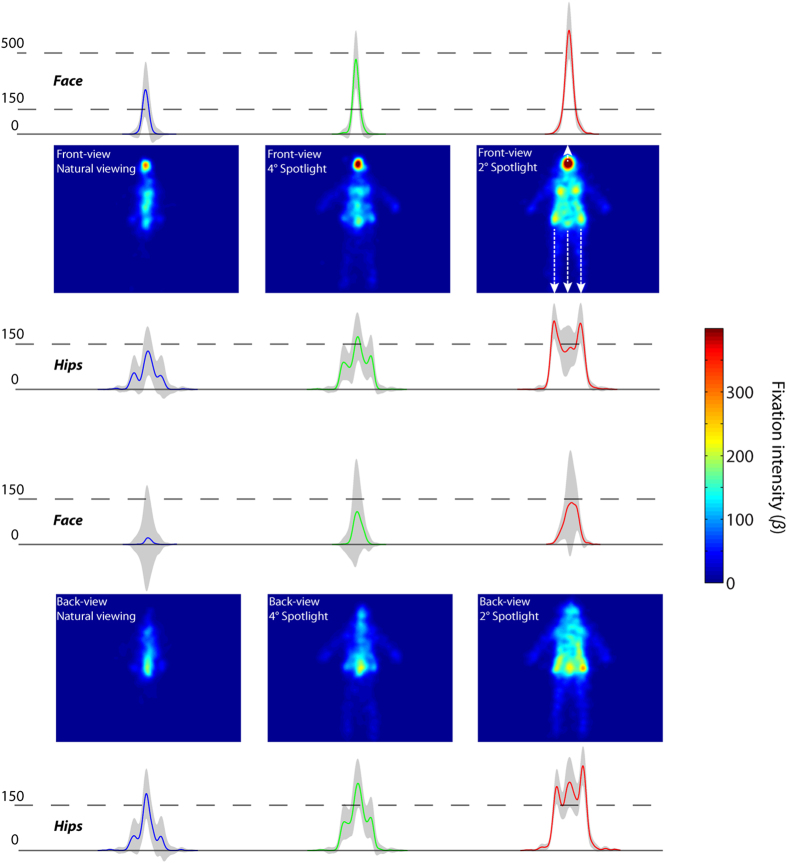
Estimated 2D coefficient (β) maps and their local maximum on the face and hip areas for each categorical predictor of the LMM (Eq. 2). Line plots of the beta values were extracted from the x axis containing the local maxima for the face and hip regions. The 95% confidence intervals are reported in the grey areas.

**Figure 4:**
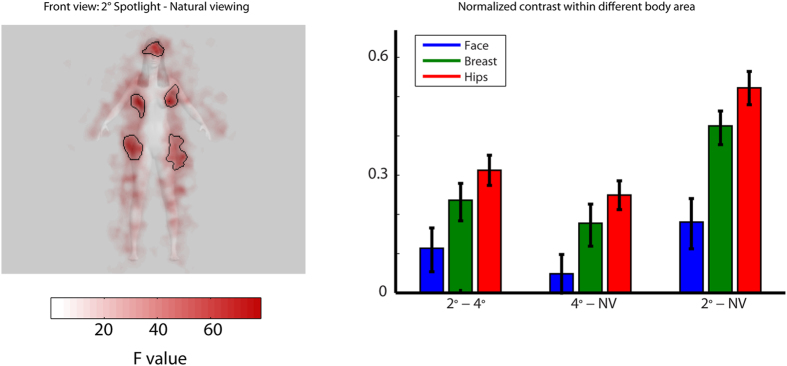
Left panel: difference fixation maps performed on the LMM (Eq. 2) between the front view 2° spotlight and natural viewing conditions. Significant clusters are outlined with black lines. Right panel: normalized contrast among the three viewing conditions within different region masks from Left panel. Error bars report standard errors. Image generated with the software MakeHuman, under a PDD licence (public domain).

To directly quantify and compare different conditions after the model fitting, we applied a profile analysis on the beta maps of the categorical predictors (Fig. 3) and carried out a post-hoc analysis within the significant area (Fig. 4). We defined they coordinate of the horizontal slices by taking the local maxima around the face and hips area of the average fixation maps. The line plots of the beta values represent the mean fixation intensity along the x axis for every horizontal slice (see Fig. 3). The areas highlighted in black in Fig. 4 are reporting the statistically significant effect resulting from the comparison between the front view natural viewing conditions with a 2° spotlight. It is worth noting that these areas are falling into meaningful bodily features: the face, the breast and the hips. A post-hoc analysis was then performed within these data-driven areas of interest.

## Additional Information

**How to cite this article**: Bovet, J. *et al*. Mapping female bodily features of attractiveness. *Sci. Rep.* **6**, 18551; doi: 10.1038/srep18551 (2016).

## Supplementary Material

Supplementary Information
