# Supplementary material for: Mapping female bodily features of attractiveness
Source: Sci Rep. 2016 Jan 21;6:18551. doi: 10.1038/srep18551 (PMC4726249; doi:10.1038/srep18551)
Supplement: Supplementary Information [file srep18551-s1.pdf]

Mapping females' bodily features of attractiveness

Jeanne Bovet, Junpeng Lao, Océane Bartholomée, Roberto Caldara, Michel Raymond

Supplementary

Table S1.

Results of the general logit mixed model for the behavioral analysis of the observers' subjective ratings. The intercept corresponds to a front view picture of a female body with an average WHR and hip size, with the face n°1, under the *natural viewing* condition.

Variable	Estimate	Confidence interval 95 %	p-value
Intercept	13.77	[11.12; 16.44]	< 0.0001
4° spotlight	0.90	[0.80; 1]	< 0.0001
2° spotlight	1.03	[0.93; 1.13]	< 0.0001
WHR	-3.43	[-4.60; -2.28]	< 0.0001
Hip size	-0.06	[-0.08; -0.05]	< 0.0001
Face n°2	-1.25	[-1.38; -1.13]	< 0.0001
Face n°3	-1.34	[-1.4; -1.21]	< 0.0001
Orientation (back)	0.03	[-0.06; 0.13]	0.53
Observer's age	0.03	[-0.03; 0.11]	0.35
Observer's income	0.06	[-0.08; 0.21]	0.41
Observer's education	0.0006	[-0.25; 0.25]	0.99

Table S2.

Results of the logit mixed models for the behavioral analysis of the observers' subjective ratings. The intercept corresponds to a front view picture of a female body with an average WHR and hip size, and with the face n°1.

Viewing condition	Variable	Estimate	Confidence interval 95 %	p-value
Natural viewing	Intercept	21.61	[18.10; 25.14]	< 0.0001
	WHR	-5.78	[-7.43; -4.13]	< 0.0001
	Hip size	-0.13	[-0.14; -0.11]	< 0.0001
	Face n°2	-1.41	[-1.59; -1.23]	< 0.0001
	Face n°3	-1.36	[-1.54; -1.18]	< 0.0001
	Orientation (back)	0.16	[0.01; 0.31]	0.045
	Observer's age	0.05	[-0.03; 0.14]	0.26
	Observer's income	0.09	[-0.08; 0.28]	0.30
	Observer's education	-0.09	[-0.39; 0.20]	0.53
4° spotlight	Intercept	12.27	[9.09; 5.45]	< 0.0001
	WHR	-3.77	[-5.28; -2.27]	< 0.0001
	Hip size	-0.05	[-0.06; -0.03]	< 0.0001
	Face n°2	-1.25	[-1.41; -1.08]	< 0.0001
	Face n°3	-1.34	[-1.50; -1.17]	< 0.0001
	Orientation (back)	0.07	[-0.06; 0.21]	0.27
	Observer's age	0.06	[-0.009; 0.14]	0.096
	Observer's income	0.003	[-0.15; 0.15]	0.96
	Observer's education	0.07	[-0.186; 0.32]	0.60
2° spotlight	Intercept	9.29	[6.02; 12.57]	< 0.0001
	WHR	-0.83	[-2.33; 0.65]	0.30
	Hip size	-0.02	[-0.03; -0.007]	0.006
	Face n°2	-1.10	[-1.27; -0.94]	< 0.0001
	Face n°3	-1.32	[-1.48; -1.16]	< 0.0001
	Orientation (back)	-0.14	[-0.28; -0.01]	0.045
	Observer's age	-0.006	[-0.09; 0.08]	0.88
	Observer's income	0.07	[-0.10; 0.25]	0.43
	Observer's education	0.01	[-0.27; 0.30]	0.90

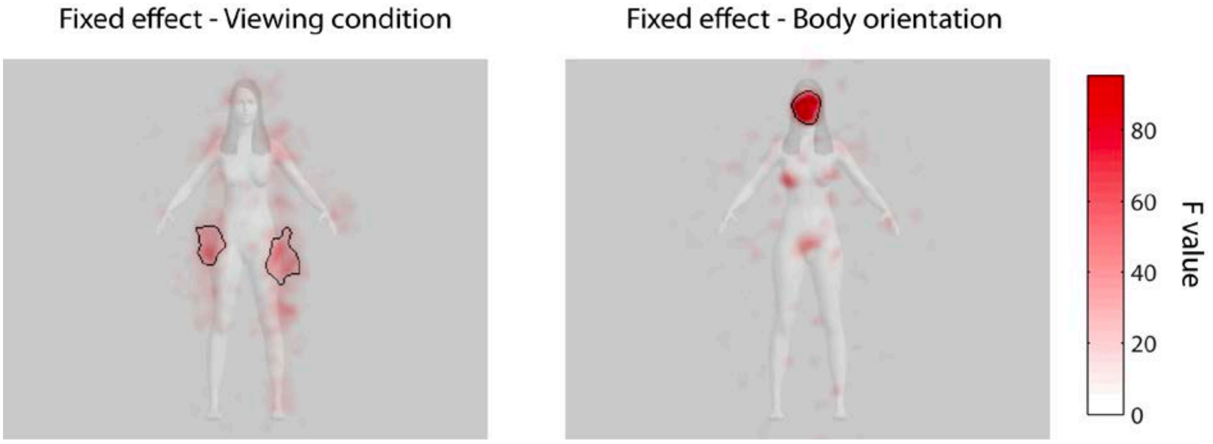

Figure S1.

ANOVA result on LMM (Eq. 1). Left panel: F-value map of the viewing condition factor. Right panel: F-value map of the body orientation factor. Significant clusters are outlined with black lines (cluster corrected $p < .05$). Image generated with the software MakeHuman, under a PDD licence (public domain).
